# Practical Aspects of ^161^Tb Production

**DOI:** 10.3390/ph19040619

**Published:** 2026-04-14

**Authors:** Marie Skálová, Tereza Janská, Matěj Štíbr, Martin Vlk, Jaroslav Šoltés, Miroslav Vinš, Sindre Hassfjell, Jiri Muller, Ján Kozempel

**Affiliations:** 1 Department of Nuclear Chemistry, Faculty of Nuclear Sciences and Physical Engineering, Czech Technical University in Prague, Břehová 7/78, 110 00 Prague, Czech Republic; 2 Centrum výzkumu Řež s.r.o., 250 68 Husinec, Czech Republic; 3 Department of Tracer Technology, Institute of Energy Technology, Instituttveien 18, 2007 Kjeller, Norway

**Keywords:** terbium-161, ^161^Tb, separation, gadolinium-160, ^160^Gd, production

## Abstract

**Background/Objectives**: Terbium-161 is an interesting and promising theranostic radionuclide, thanks to its decay characteristics (T_1/2_ = 6.95 d, E(β)_max_ = 593 keV, E(β)_av_ = 154 keV, E(γ) = 74.6 keV (10.2%)). Having similar chemical properties, it is considered as an alternative to currently used ^177^Lu. In addition, ^161^Tb emits a significant amount of conversion and Auger electrons, which contribute to the enhancement of localised therapeutic effect. The aim of this paper is to describe the preparation of ^161^Tb in quantity and quality relevant for preclinical and early clinical studies and to provide practical notes on the preparation. **Methods**: No-carrier-added ^161^Tb has been repeatedly prepared by neutron irradiation of highly enriched ^160^Gd targets (up to 98 mg of ^160^Gd_2_O_3_) at nuclear reactor LVR-15 (CV Řež, Czech Republic) in four different irradiation positions. The separation and purification process of ^161^Tb from the bulk of ^160^Gd target was performed by cation exchange chromatography with Dowex 50 W × 8 (H^+^ cycle, 200–400 mesh). Terbium-161 was obtained in ^161^TbCl_3_ form and formulated into 0.1 M HCl solution. The γ-ray spectrometry was used for radionuclide identification and radionuclidic purity and the ICP-MS method for chemical purity measurements and specific activity determination. The DOTA labelling assay was performed, as described by Gracheva et al., providing an assessment of the apparent molar activity of the preparation in terms of its competitive interaction with stable daughter nuclide ^161^Dy. **Results**: Irradiations (59.2 h to 421.52 h) of enriched ^160^Gd targets with mass ranging from 43.4 to 144.0 mg for ^160^Gd(NO_3_)_3_ and from 12.5 to 98.3 mg for ^160^Gd_2_O_3_ yielded 1.3–23.7 GBq of ^161^Tb. The separation yields of purified ^161^Tb varied from 85 to 99%, with the activities of 9.9–22.1 GBq and the highest achieved specific activity of the final product was 4.1 GBq/μg (of Tb). The DOTA chelator was radiolabelled with ^161^Tb at time points from 2 to 14 days after the end of separation (EOS). **Conclusions**: Based on our results, we describe practical aspects of terbium production at the laboratory scale with a particular focus on practical aspects and issues arising during the process that may surprise even experienced radiochemists, as lanthanoid separation is not always straightforward, even though it is well-known and has been extensively studied. The preparation of ^161^Tb in a n.c.a. form proceeds, according to the reported data, with high reproducibility and achieves significant activity levels suitable for both preclinical and clinical investigations by irradiation of highly enriched ^160^Gd targets in LVR-15 reactor with subsequent separation and purification of ^161^Tb on cation exchange resin Dowex 50 W × 8(H^+^). The produced [^161^Tb]TbCl_3_ is employed in subsequent experimental research and development for the labelling of preparations intended for preclinical applications.

## 1. Introduction

Terbium has quickly grown in popularity in recent years, being called “The Swiss army knife of nuclear medicine”, due to the existence of four clinically interesting isotopes with properties suitable for use in both diagnostic and therapeutic treatment (Table 1) [1]. It offers the possibility of using the same element for the preparation of chemically identical radiopharmaceuticals for both imaging and therapy, making it an ideal candidate for radiotheranostic application.

### 1.1. Terbium Isotopes for Theranostic Application

As mentioned above, terbium offers four clinically interesting isotopes. Terbium-155 (T_1/2_ = 5.32 d) decays by electron capture and emits gamma rays of 86.55 keV (32.0%) and 105.3 keV (25.1%); therefore, it can be considered for use in SPECT imaging with the advantage of not adding high radiation exposure to the patient. Terbium-152 (T_1/2_ = 17.48 h) emits positrons with an average energy of 1.080 MeV (17%) and would be suitable for PET imaging. Terbium-149 (T_1/2_ = 4.12 h) emits short-range alpha-particles at an energy of 3.967 MeV (16.7%) suitable for targeted alpha therapy (TAT), and positrons with an average energy of 730 keV (7.1%) which enables PET imaging.

Terbium-161 (6.95 d) emits low energy β^−^ particles with an average energy of 154 keV (101%) and gamma rays of 74.6 keV, which are suitable for SPECT imaging. Terbium-161 is an analogue of currently used ^177^Lu (T_1/2_ = 6.65 d, Eβ^−^_average_ = 134 keV) in terms of both chemical and physical properties. However, ^161^Tb emits a significant amount of conversion and Auger electrons in contrast to ^177^Lu, which increases its potential therapeutic efficacy. The advantage of the chemical similarity of these elements is the possibility of using radiolabelling chemistry techniques already established for ^177^Lu while maintaining the pharmacokinetic profile of any targeting agent with a DOTA chelator [4].

### 1.2. Terbium-161 Across the Time

The first mentions of ^161^Tb with high specific activity can already be found in the 1960s, when Bhatki et al. prepared a high specific activity source of ^161^Tb from Gd for studying the recoil-less emission and absorption of the 26 keV gamma ray of ^161^Dy [5]. De Jong et al. evaluated [^161^Tb]Tb-DTPA-octreotide in vitro and in rats in comparison with [^111^In]In-DTPA-octreotide, as a somatostatin analogue with potential for intraoperative scanning and radiotherapy in 1995 [6]. Fischer et al. presented a comparison of ^177^Lu and ^161^Tb labelled monoclonal antibody chCE7 for radioimmunotherapy in 2009 [7]. Lehenberger et al. proposed the possibility of using ^161^Tb as an alternative to ^177^Lu for targeted radionuclide therapy in 2011, being able to produce n.c.a. ^161^Tb by irradiating highly enriched ^160^Gd targets and use the obtained ^161^Tb for the preparation of [^161^Tb]Tb-DOTA-Tyr^3^-octreotate [8]. Müller et al. published an in vivo proof-of-concept study of the production of all four terbium radioisotopes in 2012, testing them in parallel with tumour bearing mice using a folate receptor targeting agent [2]. In 2014, Müller et al. compared the effects of ^161^Tb and ^177^Lu in a study using a DOTA-folate ligand and Grünberg et al. investigated the efficacy of anti-L1CAM radioimmunotherapy using mAb chCE7 labelled with ^177^Lu or ^161^Tb [9,10]. Haller et al. investigated the contribution of Auger/conversion electrons to renal side effects after radionuclide therapy in 2016, performing a preclinical comparison of [^161^Tb]Tb-folate and [^177^Lu]Lu-folate [11]. Müller et al. used ^161^Tb to label PSMA-617 to perform a preclinical comparison with [^177^Lu]Lu-PSMA-617 and demonstrated that [^161^Tb]Tb-PSMA-617 was more effective in the killing of tumour cells in vitro compared to [^177^Lu]Lu-PSMA-617 [4].

### 1.3. Clinical Trials with Terbium-161

All these steps led to the first-in-humans application of ^161^Tb in 2021—Baum et al. investigated the feasibility of visualising the physiologic and tumour biodistributions of [^161^Tb]Tb-DOTATOC and confirmed that the emitted γ-radiation of ^161^Tb can be used for whole-body planar as well as SPECT/CT imaging of even low activities of injected ^161^Tb [12]. In the time of writing, seven clinical trials with ^161^Tb are ongoing (Table 2).

### 1.4. Production of Terbium-161

The most convenient method for no-carrier-added ^161^Tb production at high specific activities is the method proposed by Lehenberger et al. [8]. It is already well known, but briefly, ^161^Tb is produced by the neutron irradiation of highly enriched ^160^Gd target via short-lived intermediate ^161^Gd (3.66 min), which decays to ^161^Tb.

The increasing interest in ^161^Tb and prospects of insufficient reactor production of radionuclides in the future led to the investigation of cyclotron production route for ^161^Tb, namely, deuteron-induced reactions ^160^Gd (d,x) ^161^Tb on Gadolinium target by Tárkányi et al. in 2013 [20]. However, even when using highly enriched ^160^Gd target and short irradiations, large amounts of ^160^Tb (T_1/2_= 72.34 d) are produced besides the ^161^Tb via the disturbing reaction ^160^Gd(d,2n)^160^Tb (cross sections 2–3 times higher than for the ^161^Tb production route). Roughly, two times more of ^160^Tb than ^161^Tb nuclei would be present in the irradiated sample, making this an unsuitable production route for medical purposes.

### 1.5. Separation and Purification Process

Ion exchange chromatography is the most important and reliable technique for the separation of lanthanides and has been used with 2-hydroxyisobutyric acid (α-HIBA) as the eluent since the 1950s [21]. Therefore, it is also the most common method used for separation of the prepared ^161^Tb from the bulk irradiated target (several of them compared in Table 3). After the column is loaded with the dissolved target, the lanthanides are eluted sequentially from higher to lower atomic numbers using different concentrations of α-HIBA. In the Gd/Tb system, ^161^Tb is first eluted using 0.13 M α-HIBA, followed by Gd using 0.5 M α-HIBA. If Dy is present, it is eluted even before ^161^Tb, also using 0.13 M α-HIBA [8,22]. However, a procedure has also been described for the separation of these three elements using three different concentrations of α-HIBA: 0.14 M for the elution of Dy, 0.2 M for the elution of Tb, and finally 0.5 M for Gd [23]. The separation step is followed by the purification and concentration of ^161^Tb on a second smaller column filled with the same cation exchange resin or with (bis(2,4,4-tri-methyl-1-pentyl) phosphinic acid extraction resin—LN3 resin [22]. Holiski et al. reported a cation-exchange separation followed by the concentration on DGA resin (0.01 M HNO_3_) with α-HIBA removal, a secondary fine separation using LN resin (di(2-ethylhexyl)phosphoric acid extraction resin) with 0.5 M and 0.75 M HNO_3_, and a final concentration on DGA resin, from which concentrated, pure ^161^Tb was stripped using 0.1 M HCl.

Another option of ^161^Tb separation from gadolinium target is extraction chromatography using resins containing acidic extraction agents based on alkylphosphonates impregnated onto an inert polymer support as the stationary phase, and nitric acid at various concentrations as the mobile phase (several of which are compared in Table 4). In this case, the elements are eluted in the reverse order compared to a cation exchange. Thus, Gd is first eluted using a lower concentration of HNO_3_ followed by ^161^Tb using a higher concentration of HNO_3_. An initial crude separation is usually followed by secondary purification and concentration on another column.

When comparing these two separation methods, cation exchange chromatography appears to be more practical, as the cation exchange resins have a higher loading capacity, making the process more scalable. Furthermore, there is a risk of ^161^Tb product contamination by organophosphates possibly released from the extraction resins as well as significant Gd tailing. Therefore, we propose that extraction methods may be more advantageously applied as a secondary separation step for the purification and concentration of the ^161^Tb product following the initial separation. As mentioned earlier, ^161^Tb is a very promising radionuclide, especially as an alternative to currently used ^177^Lu. Although its successful production is occurring in a growing number of facilities worldwide, ^161^Tb production still must be upscaled to provide enough activity for clinical studies, which is unfortunately complicated by the lack of availability of the highly enriched ^160^Gd target material. This issue should hopefully improve soon, as many efforts are being established to produce more stable enriched isotopes, making them more available in the future. More investments and developments in scaling ^160^Gd production are ongoing, for example, by ASP Isotopes, Kinectrics, TMC2, Orano, SHINE, Nusano, etc. [27]. Terbium-161 is also currently commercially available from TerThera and Isotopia.

The aim of this study is to develop a method for the production of ^161^Tb from an enriched ^160^Gd target, including its subsequent separation and purification, enabling the repeated production of ^161^Tb in a form, quantity and quality suitable for preclinical testing, and thus contribute to global ^161^Tb production. Based on the experience gained during this research, practical notes on the production process are also provided.

## 2. ^161^Tb Preparation Suggestions

### 2.1. Target Material—Highly Enriched Gd_2_O_3_

Terbium-161 can be produced as n.c.a. by neutron irradiation of ^160^Gd. Since natural Gd contains only 21.66% of ^160^Gd, a highly enriched material is required, and can be purchased as highly enriched ^160^Gd_2_O_3_. However, the availability of the enriched material is scarce; hence, valuable. Therefore, it is advantageous or even necessary to recycle and reuse already irradiated targets. The recovered gadolinium can be obtained by evaporation of the gadolinium fractions (from separation) to dryness followed by calcination to eliminate organic impurities (α-HIBA) and to convert the gadolinium to the oxide form. From our experience, the processing of the recycled targets resulted in a ^161^Tb sample with higher specific activities, because the ^159^Tb isotope is separated out together with ^161^Tb. The repeated irradiation of recycled ^160^Gd targets also results in gradual burning of ^158^Gd isotope because of its high neutron cross-section (2.15 b), leading to declination of ^158^Gd/^160^Gd ratio. This results in more pure ^161^Tb product with higher specific activities, because less ^159^Tb is produced (via ^158^Gd (n, γ) ^159^Gd(β^−^, 18.479 h) ^159^Tb). A comparison of the specifics and differences between two target forms is shown in Table 5.

### 2.2. Preparation of the Target

The target material must be enclosed in a quartz ampoule that has no damage or cracks. The tightness of the ampoule can be verified by immersion in water or under UV light. The sealing of ampoule requires a certain hand-mindedness to ensure that no impurities are introduced, and the ampoule is properly sealed. The target material can be used as purchased (oxide form) or converted to nitrate form by dissolution in ultrapure concentrated HNO_3_ and evaporation to dryness (several times). Each option has its advantages and disadvantages, which are compared in Table 6. The target can also be prepared by using gadolinium recycled from previous experiments (Table 5). Indeed, the compound will still be in an oxide (or nitrate) form.

The main disadvantage of a target in nitrate form arises from its hygroscopic properties and content of crystalline water, as it readily forms hydrates. As a result, the ampoule is more prone to failure or crack during irradiation and subsequent opening, due to possible overpressure from water vapour or gases generated inside the ampoule during the irradiation in the nuclear reactor (including the moisture retained in the gadolinium nitrate). Moreover, nitrate compounds may decompose to form volatile NO_x_ species, further contributing to increased pressure within the ampoule. Ampoule failure can lead to complications such as target loss, and therefore loss of the product (^161^Tb), as well as contamination of the reactor pool.

### 2.3. Irradiation in LVR-15 Nuclear Reactor

**Figure 1:**
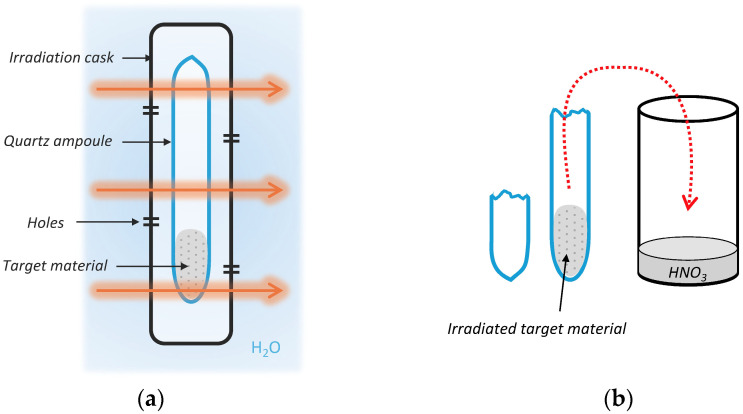
(**a**) Neutron irradiation of the target material in LVR-15 nuclear reactor. The target material is sealed in quartz ampoule and put in an irradiation cask. (**b**) Opening of the ampoule and dissolution of the irradiated target material in HNO_3_. The target can be dissolved directly in the ampoule and transferred to a flask by pouring or using a pipette. Alternatively, it can be poured into the flask while still dry; however, the ampoule usually needs to be rinsed afterward.

A schematic illustration of irradiation of the ampoule containing the target material and subsequent opening and dissolution of the irradiated target are shown in Figure 1. The resulting irradiation yield (and therefore the activity of prepared ^161^Tb) depend on a combination of many factors, naturally on the amount of target material and time of irradiation. However, the maximum advantageous time of irradiation is about 14 days, as the activity will not increase much further. Longer irradiation time also leads to higher accumulation of relatively long-lived ^160^Tb (via the ^159^Tb(n,γ)^160^Tb reaction) and production of ^159^Tb (via ^157^Gd(n,γ) ^158^Gd followed by ^158^Gd(n,γ) ^159^Gd (β^−^, 18.479 h) →  ^159^Tb), which decreases the specific activity and contributes to ^160^Tb formation. At the same time, burn-up of ^157^Gd, a strong neutron absorber, alters the neutron economy during irradiation and can affect reaction pathways and product yields. Furthermore, prolonged irradiation results in greater accumulation of the stable daughter product ^161^Dy due to the decay of ^161^Tb, leading to a significant reduction in product quality [8]. However, ^161^Dy can be effectively separated if the method is sufficiently optimised, whereas ^160^Tb reduces the final radionuclide purity.

Other factors affecting the irradiation yield are position in nuclear reactor, neutron flux at the given position, but also thickness and type and shape of the irradiation ampoule and type of irradiation cask, which can be tight (waterproof) or drilled (allows cooling of the ampoule by the water of the primary circuit). However, not all reactors allow for the use of the drilled cask.

### 2.4. Opening of the Ampoule

Prior to opening, ampoules containing nitrate-form targets should be cooled (e.g., using dry ice) to mitigate the risk of overpressure-induced rupture due to the accumulation of vapours or gases generated within the sealed ampoule during irradiation. The outer surface of the ampoule should be rinsed (with ultrapure acid and ultrapure water) to remove any impurities potentially present on the outer surface of the ampoule, to prevent contamination of the irradiated target material. Opening of the ampoule can be done either by cutting of the top of the ampoule or crushing it into small pieces. The main differences and advantages are listed in Table 7.

### 2.5. Dissolution of the Irradiated Target

Based on our experience, the nitrate form of the target material can be easily processed, as it is possible to dissolve in less concentrated acid without heating. However, the solubility is more complicated if organic impurities are present, and the volume of the dissolved target is also larger, which is not practical for column loading. The processing of the target in nitrate form also involves the already mentioned disadvantages of potential overpressure of the ampoule.

The processing of the target in oxide form involves dissolution in concentrated HNO_3_ (with heating), evaporation to dryness, and redissolution in ultrapure water. A dissolution of irradiated target in 3.2% HNO_3_ solution was also performed in several of our experiments, but the dissolution time and the volume of final solution of dissolved target were higher (2–3.5 mL), which was not practical for column loading, as was the case with the targets in nitrate form. The ideal conditions for loading the target solution include using a minimal solution volume, maintaining the correct pH (slightly acidic), and performing the loading slowly (e.g., gravimetrically).

### 2.6. Separation

**Figure 2:**
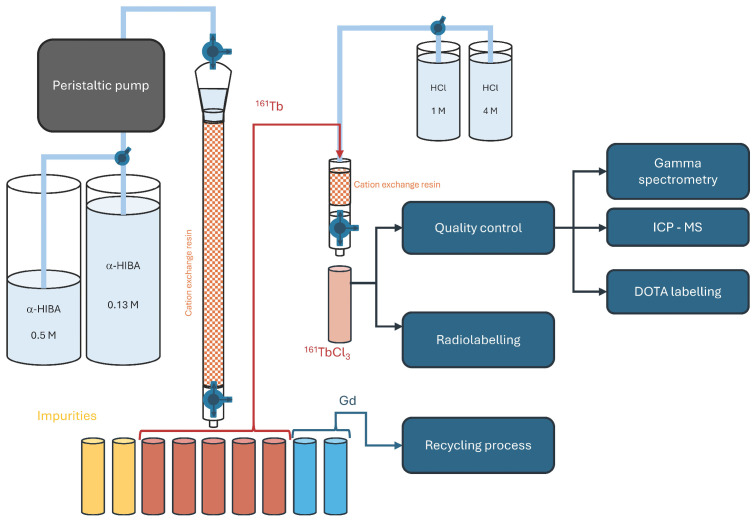
Scheme of the separation and purification process of ^161^Tb. The dissolved irradiated target is loaded on the column filled with 3 mL of Dowex 50 W × 8 (H^+^, 200–400 mesh) transferred to NH_4_^+^ form. Impurities and ^161^Tb are eluted by 0.13 M α-HIBA, Gd is eluted by 0.5 M α-HIBA. The volume of α-HIBA in both concentrations varies depending on the target mass, but the volumes are usually between 80 and 170 mL for 0.13 M α-HIBA and between 10 and 20 mL for 0.5 M α-HIBA. Terbium-161 containing fractions are acidified and loaded on the second smaller column with 0.5 mL of Dowex 50 W × 8 (H^+^, 200–400 mesh), purified from α-HIBA (by 9 mL of 1 M HCl) and eluted in ^161^TbCl_3_ form by 5 to 10 mL of 4 M HCl (gravimetrically). Eluted Gd is recovered and prepared for another irradiation. The colours of the tubes and solutions are for illustrative purposes only.

The most common method used for separation of ^161^Tb from the bulk irradiated target is cation exchange chromatography with the 2-hydroxyisobutyric acid (α-HIBA) as an eluent. A diagram of such separation followed by a purification process is shown in Figure 2. The elution time of ^161^Tb can vary a lot, as it is affected by many parameters:

- Dimensions of separation column, bed volume;
- Flow rate;
- Type of sorbent used for separation, e.g., Dowex 50 W × 8 (H^+^ form, 200–400 mesh) transferred to NH_4_^+^ form (this study)—the details of the method used for transferring to NH_4_^+^ form are described in Section 4, Materials and Methods;
- Concentration of α-HIBA solution, usually 0.13 M for ^161^Tb elution and 0.5 M for Gd elution;
- pH of the α-HIBA solution;
- Mass of the target;
- Amount and type of impurities—the higher the amount (mass) of impurities, the bigger the retardation of elution peaks.

The separation process can be performed with manual fraction collecting even when processing high activity targets (up to 20 GBq) (with adequate shielding) due to the short range of particles emitted by ^161^Tb, but an automated fraction collector is recommended to lower the radiation exposure of the workers. A peristaltic pump can be used to enhance the separation process—to ensure the constant flow of eluents.

This semi-automated process offers the advantages of automation (same fraction volumes, constant flow, etc.) and still allows for active intervention according to the situation (e.g., changing of eluent concentration), which is useful because the separation process (elution profiles) is hardly the same each time, even when processing similar amounts of targets.

### 2.7. Purification and Formulation of ^161^TbCl_3_

Once ^161^Tb has been separated from the bulk target material and impurities, a purification step is required to remove α-HIBA. This is followed by the concentration and formulation of ^161^Tb into a solution suitable for radiolabelling.

Due to the potential overlap of ^161^Tb elution peak tails with Gd or impurities elution peaks, it is recommended to discard initial and final fractions containing ^161^Tb for purification (omit fractions from the edge of the peak) to ensure the final solution contains almost no impurities. Although this step reduces the ^161^Tb production yield, it allows for obtaining higher purity and specific activity. The radionuclidic purity of ^161^Tb fractions chosen for purification should be verified by gamma spectrometry.

The purification step can be performed on the same cation exchange resin, which was used for the separation process, but in H^+^ form and with hydrochloric acid as an eluent. The ^161^Tb fractions from the first separation column are acidified to a pH of 1 to break the α-HIBA complex and enable efficient loading onto the secondary strongly acidic column. The column is washed with 1 M HCl to remove residual α-HIBA, and ^161^Tb is subsequently eluted as [^161^Tb]TbCl_3_ using 4 M HCl. A disadvantage of this method is the large volume and high acidity of the eluate, which necessitates evaporation and reconstitution of the prepared [^161^Tb]TbCl_3_ to achieve the desired radioactivity and acid concentration suitable for radiolabelling. An option for ^161^Tb concentration without evaporation is the use of LN3 resin, which allows for washing out ^161^Tb in ^161^TbCl_3_ form with 0.05 M HCl directly in a small volume and pH suitable for radiolabelling [22].

### 2.8. Quality Control

#### 2.8.1. Radioactivity Measurements

Terbium-161 is a low-energy gamma emitter—more than 99% of its emitted gamma and X-rays have an energy below 100 keV; therefore, it is important to use the appropriate calibration factor not only for the distance of the measured sample from the detector, but also for all other different geometric conditions, such as the type of container (e.g., different types of glass vials, Eppendorf tubes, syringes) or filling volume. The precise activity measurements of ^161^Tb using an ionisation chamber were published by Juget et al. [28].

Besides the measurements of the total activity of the sample (with appropriate calibration factor), gamma spectroscopy is required to determine radionuclidic impurities. The main one is ^160^Tb (T_1/2_ = 72.3 d; E_γ_ = 879.38, I = 30.1%) and to verify that the prepared ^161^Tb sample is in a quality suitable for potential use in medicine, the presence of ^160^Tb needs to be determined after the decay of ^161^Tb. This was highlighted, e.g., in [^161^Tb]Tb-DOTATOC production by Favaretto et al. [29]. Other impurities can be ^169^Yb (E_γ_ = 197.96 keV, I = 35.93%; usually eluted before ^161^Tb) and gadolinium, which can be measured by its isotopes ^153^Gd (E_γ_ = 97.43 keV, I = 30.0%) and ^159^Gd (E_γ_ = 363.54 keV, I = 11.78%). In some cases, ^153^Sm (E_γ_ = 103.18 keV, I = 29.14%) as an impurity was observed.

#### 2.8.2. Chemical Purity Measurements

The presence of stable impurities in samples taken after the purification process can be measured by the inductively coupled plasma mass spectrometry (ICP-MS) method [26]. It also allows for determining the specific activity of the prepared ^161^Tb solution. When measuring samples by ICP-MS, it is necessary to wait until the activity drops to a negligible value, or to adapt the method to measure radioactive samples.

Terbium can be determined by its isotope ^159^Tb, which is also the only stable isotope of terbium. 

The presence of dysprosium can also be measured, but since ^161^Dy is a daughter product of ^161^Tb, its quantity depends on the efficiency of the separation process as well as on the amount and decay time of ^161^Tb sample taken for the ICP-MS measurements. Another complication of this determination is the isobaric interference of these two radionuclides. Therefore, it is important to know the end of separation time in order to calculate the amounts of ^161^Dy and ^161^Tb at a given time, assuming that no ^161^Dy is present in the ^161^Tb samples immediately after separation.

McNeil et al. used NH_3_ gas to shift the mass of ^161^Tb ions to a different mass (^161^Tb^+^ → ^161^TbNH^+^ (M + 15), ^161^Tb^+^ → ^161^TbNH(NH_3_)^+^ (M  +  32)) and eliminate interference from ^161^Dy, thus allowing the chemical purity of the final product to be analysed before decay. The mass shift M + 32 provided a more accurate representation of ^161^Tb content when compared to gamma spectroscopy [26].

#### 2.8.3. DOTA Labelling

The radiolabelling of DOTA molecule with ^161^Tb followed by thin layer chromatography (TLC) can be performed as a quick verification of the quality of the pure ^161^Tb stock solution, as described by Gracheva et al. [22]. The yield of radiolabelling corresponds to ^161^Tb half-life—it decreases with time, due to the ^161^Tb conversion to the daughter product ^161^Dy, which competes with ^161^Tb for binding. It can also be used to determine the age of purchased ^161^Tb stock solution.

## 3. Results and Discussion

**Figure 3:**
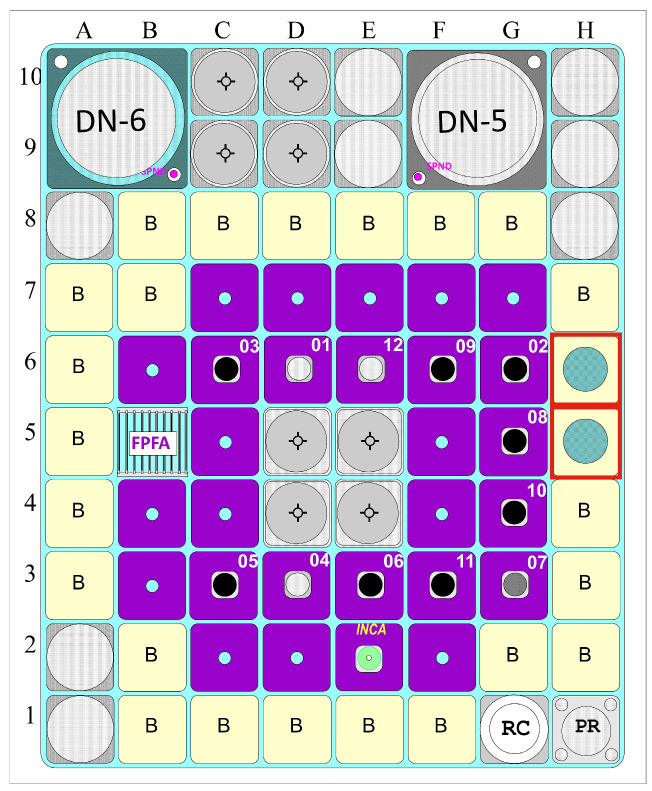
Reactor core configuration of the LVR-15 research reactor. The irradiation channels used for ^161^Tb production were H5 and H6 (marked with red squares).

In this paper, we report 23 targets, which were prepared at FNSPE and irradiated in LVR-15 reactor (CV Řež, Czech Republic; reactor core configuration is shown in Figure 3). Irradiations were performed in vertical channels. The two standard irradiation channels for radioisotope production are designated H5 and H6, each containing four irradiation slots (H5/1–H5/4 and H6/1–H6/4). The channels are accessible via a remotely controlled loading and unloading system, which allows for the insertion and extraction of irradiation capsules at virtually any time during the reactor operation. Terbium-161 was produced in positions H5/2, H5/3, H6/2, and H6/3.

### 3.1. Calculation of Neutron Fluxes and Specific Activity of ^161^Tb

As a part of the preparations for ^161^Tb production in LVR-15 research reactor, the neutron fluxes achievable in vertical irradiation channels H5 and H6 in positions used for ^161^Tb production (H5/2, H5/3, H6/2 and H6/3), were calculated and are presented in Table 8. The table includes four neutron energy intervals and a “total” value, which is the sum of the first three intervals in each column (excluding the fourth). The energy intervals cover 0–0.5 eV, 0.5 eV–0.1 MeV, 0.1 MeV–20 MeV. The third and fourth intervals partially overlap and are presented separately because, for certain types of reactor experiments involving material irradiation, both values are relevant.

**Figure 4:**
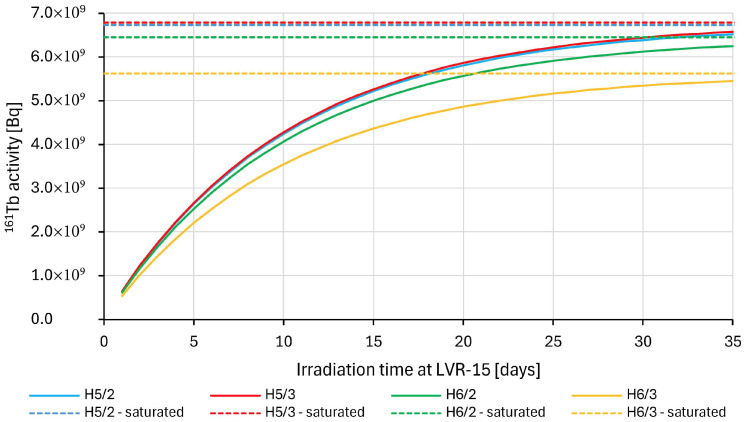
Calculated dependence of ^161^Tb activity (from 30 mg of enriched Gd_2_O_3_ target (98.2 ± 0.1% ^160^Gd)) on irradiation time in different vertical irradiation positions (H5/2, H5/3, H6/2, H6/3) at nuclear reactor LVR–15. Neutron fluxes achievable in these positions are listed in Table 8.

Then, an activity of ^161^Tb produced by irradiation of 30 mg of enriched ^160^Gd_2_O_3_ target (98.2 ± 0.1% ^160^Gd) related to the irradiation time in each of these positions was calculated (Figure 4). As can be seen, the position in the reactor significantly affects the resulting specific activity of produced ^161^Tb, with the most advantageous irradiation being in position H5/3 and H5/2. The maximum favorable time of irradiation is about 14 days, as the activity will not increase much further and a longer irradiation leads to higher accumulation of ^160^Tb, stable daughter product ^161^Dy and ^159^Tb (via ^158^Gd activation), causing a significant reduction in specific activity. The cost of irradiation also significantly increases with time.

### 3.2. Target Preparation and Irradiation

The irradiated targets are marked chronologically and with the serial number of ampoules in the given irradiation. For example, the first reported irradiation includes two targets, first in oxide form (labelled as 1_1), second in nitrate form (1_2).

Targets were prepared and irradiated in both oxide and nitrate forms, their mass ranged between 43.4 mg and 144.0 mg for ^160^Gd(NO_3_)_3_ and between 12.5 mg and 98.3 mg for ^160^Gd_2_O_3_. Parameters of irradiation and prepared targets are listed in Table 9. However, due to minor complications associated with the nitrate form of the target, as described in Section 2.2 about the preparation of the target, we continued to prefer only targets in the oxide form.

Irradiation of two groups of smaller targets (four ampoules containing 12.5 mg of ^160^Gd_2_O_3_—targets 5_(1–4) and 6_(1–4)) followed by dissolution and processing of these targets together were performed. Although irradiating several smaller targets seems more advantageous in terms of the resulting activity relative to the irradiation time, the processing of several smaller targets was not so effective, mostly because of the time needed for completion of irradiated targets, their dissolution and the increased potential for losses during handling. Therefore, we prefer the irradiation of larger amounts of target material at once.

### 3.3. Separation and Purification Process

All the irradiated targets were processed to obtain pure ^161^Tb samples; however, we report only those separations for which complete analytical data were available (ICP-MS measurements and determination of the specific activity of the final ^161^Tb sample), and only for targets in oxide form (Table 10).

**Figure 5:**
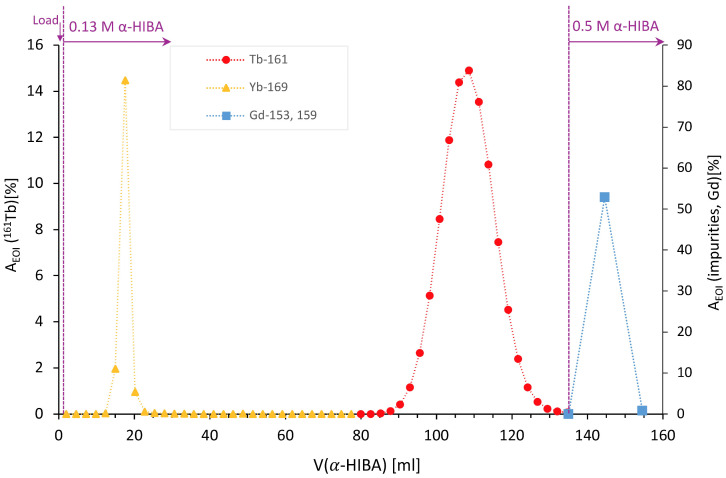
Elution profile of target 8_2 (49.8 mg); c(α-HIBA): 0.13 M, after 134.6 mL: 0.5 M; flow rate 0.2 mL/min. The main axis represents A_EOI_ of ^161^Tb (in %), A_EOI_ of impurities and gadolinium (in %) are shown on the secondary (right) axis.

To represent the general results, an elution profile for the separation of ^161^Tb from target 8_2 (49.8 mg) is presented in Figure 5. The impurities, mainly ytterbium, were eluted first with 0.13 M α-HIBA and ^161^Tb began to elute from the column after circa 80 mL of 0.13 M α-HIBA. Gadolinium was eluted with 20 mL of 0.5 M α-HIBA in two fractions. This separation system—a chromatographic column with 150 × 5 mm dimension filled with 3 mL of the resin Dowex 50 W × 8 (H^+^) (200–400 mesh) in NH_4_^+^ form—was tested for up to 98.3 mg of target mass at a time (target 8_1). The volume of α-HIBA needed to wash out the ^161^Tb and Gd varies depending on the mass of the target, pH of eluent and amount of impurities.

### 3.4. Dechelatation and Purification of ^161^Tb Containing Fractions

**Figure 6:**
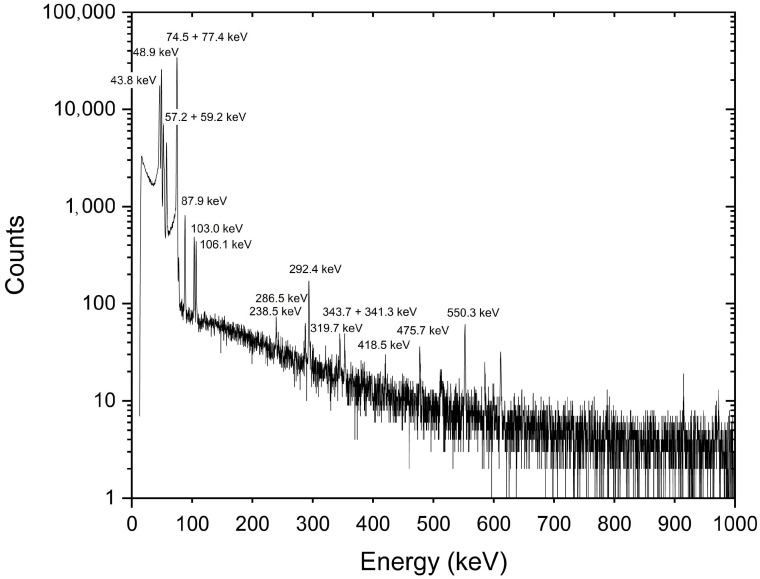
Gamma spectrum of ^161^Tb sample obtained after the separation and purification process.

Not all the fractions containing ^161^Tb-α-HIBA complex were always purified to ensure the final solution contains almost no impurities. Although this step reduces the final ^161^Tb yield, it allows for obtaining higher chemical and radionuclidic purity; hence, yielding a higher apparent molar activity. The radionuclide purity of ^161^Tb fractions chosen for purification was verified by gamma spectrometry (Figure 6). Since the ^161^Tb purified of α-HIBA was eluted from the secondary column in the form of [^161^Tb]TbCl_3_ with 4 M HCl, it comes in highly acidic solution. Therefore, it was evaporated and reconstituted in 0.05 M HCl solution, for better use in next experiments, mainly radiolabelling. Although purification on cation exchange resin is reliable and effective, we plan to incorporate the purification and concentration of ^161^Tb using LN3 resin, from which ^161^Tb can be eluted directly in a small volume with 0.05 M HCl. This approach avoids handling multiple fractions in larger volumes (and the associated potential product losses) as well as the need to evaporate the radioactive solution. On the other hand, the use of LN3 resin introduces a potential risk of contaminating the final product with organophosphates released from the resin.

For comparison, the highest final yield of reported purified ^161^Tb samples was 99.47% (6_(1–4)), but it contained Gd in a concentration of almost 1 mg/mL (Table 11) and had a lower apparent activity; therefore, we preferred to get lower, but still high yields (85–93%) of ^161^Tb with higher purity of the final product.

### 3.5. Radiometric Measurements

The radioactivity of all samples was measured by the CRC-55tW detector. In the first experiments, all the fractions were also measured by gamma spectrometry on an HPGe detector (ORTEC^®^ DSPEC jr. 2.0 ™) to analyse the radionuclidic composition. Once the method was developed and established, only ^161^Tb containing fractions were verified. The main impurity washed before ^161^Tb was identified as ^169^Yb. Gadolinium was measured by its isotopes ^153^Gd (E_γ_ = 97.43 keV, I = 30.0%) and ^159^Gd (E_γ_ = 363.54 keV, I = 11.78%) and it contained ^153^Sm as an impurity in some cases (E_γ_ = 103.18 keV, I = 29.14%). The presence of ^160^Tb (T_1/2_ = 72.3 d; E_γ_ = 879.38, I = 30.1%) was determined after the decay of ^161^Tb (decay time at least 4–5 half-lives of ^161^Tb), to verify that the prepared ^161^Tb sample would be in a quality suitable for potential use in medicine. The radionuclide purity of ^161^Tb samples was always ≥99.999%.

### 3.6. Chemical Purity and Specific Activity Determination

The specific activity of ^161^Tb was determined from samples collected for ICP-MS measurements. It was calculated as the activity of the sample (corrected to EOI) divided by the amount of ^159^Tb present (mass in μg), which was measured by ICP-MS. Currently, there is inconsistency among laboratories worldwide in expressing the specific or molar activity of ^161^Tb. The nuclide is reported variously as apparent molar activity in MBq/nmol (e.g., [^161^Tb]Tb-DOTA [24] or [^161^Tb]Tb-crown-αMSH [26]), as radioactivity concentration in MBq/μL [22], or as specific activity relative to total terbium mass (e.g., GBq/μg of Tb in this study, TBq/mg [8]). Given the growing demand for terbium, it would be advantageous for laboratories to adopt a uniform method for reporting the resulting ^161^Tb product.

#### 3.6.1. ICP-MS Analyses

The presence of stable impurities in the final ^161^Tb solution was measured by ICP-MS method on Agilent system 7500 in multi-element mode without collision program. To calculate the specific activity of final ^161^Tb solution, the amount of stable ^159^Tb impurity determined by ICP-MS was used. Dysprosium-161 and other isotopes of Dy were also measured, but since ^161^Dy is a daughter product of ^161^Tb, its quantity depends on the amount and age of ^161^Tb sample taken for the ICP-MS measurements. Although the samples were measured after the ^161^Tb decay, any residual presence of ^161^Tb could also have contributed to the 161 signal. The measured amount of Dy is therefore not reported. Other elements are listed in Table 11.

All stable isotopes of gadolinium were measured. The total gadolinium concentration was then determined as the sum of the measured concentrations of all Gd isotopes.

As can be seen in Table 10 and Table 11, the ^161^Tb sample obtained from the processing of targets prepared directly from the purchased oxide (6_(1–4), 9_1, 12_1) contained more impurities and had significantly lower chemical purity than other processed targets, which were prepared from the gadolinium recycled from the previous experiments.

#### 3.6.2. DOTA Labelling

**Figure 7:**
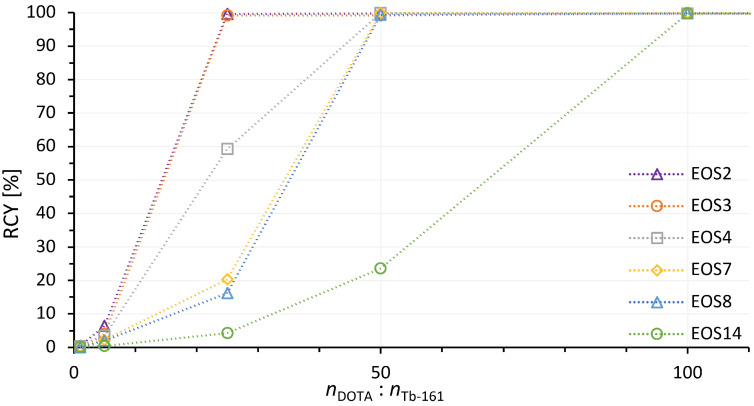
Radiochemical yield (RCY [%]) of DOTA with ^161^Tb (from target 8_2) at different DOTA to ^161^Tb molar ratio (*n*_DOTA_:*n*_161Tb_).

The DOTA radiolabelling experiments were performed to verify the quality of the pure ^161^Tb stock solution and because of current and future experiments with radiolabelling of DOTA bearing vectors. The radiolabelling was repeated six times during 14 days after the end of separation (EOS) of ^161^Tb. For the demonstration, results from target 8_2 are shown in Figure 7. The yield of radiolabelling DOTA molecule corresponds to ^161^Tb half-life—it decreases with time, due to the ^161^Tb conversion to the daughter product ^161^Dy, which competes with ^161^Tb for binding.

## 4. Materials and Methods

Terbium-161 was produced by the indirect production route firstly proposed by Lehenberger et al. [8]. For this purpose, highly enriched gadolinium oxide (Gd_2_O_3_, 98.2 ± 0.1% ^160^Gd) was purchased from Isoflex, (San Francisco, CA, USA). The isotopic composition of the material and its chemical admixtures are shown in Table 12 and Table 13. Ultrapure water was prepared by MilliQ. HNO_3_ (NORMATOM^®^, Ultrapure for trace metal analysis), HCl (NORMATOM^®^, Ultrapure for trace metal analysis) and Multi-Element Quality Control Standards 1 and 2 (VWR^®^ ARISTAR^®^) were purchased from VWR International s.r.o. (Stříbrná Skalice, Czech Republic). Dowex 50 W × 8 (H^+^) (200–400 mesh, Supelco^®^), α-HIBA (99%, Sigma-Aldrich), TLC silica gel plates 60F_254_ (Supelco^®^), Ammonium acetate (for molecular biology, ≥ 98%, Sigma Aldrich) and Methanol (CHROMASOLV^®^ for HPLC, ≥99.8%) were purchased from Sigma-Aldrich (Merck, Prague, Czecg Republic). DOTA was purchased from CheMatech (Dijon, France) and Ammonium hydroxide solution (analytical grade) from Lach-Ner (Neratovice, Czech Republic).

The resin Dowex 50 W × 8 (H^+^ form, 200–400 mesh) was prepared and transferred to NH_4_^+^ as follows: The new dry resin was washed with ultrapure water several times and left to swell in ultrapure water for several hours (usually overnight). The swelled resin was then stirred with 1 M solution of NH_4_OH for at least 1 h, usually longer. The separation column was filled with 3 mL of resin and subsequently washed with ultrapure water until the pH of the eluate was 7 (usually circa 50 mL of ultrapure water).

### 4.1. Target Preparation and Irradiation

The targets were prepared by sealing the appropriate amount of highly enriched Gd_2_O_3_ (98.2 ± 0.1% ^160^Gd) or transforming the purchased oxide to nitrate form (^160^Gd(NO_3_)_3_) and then sealing in quartz ampoules. Irradiations of targets were performed in the nuclear reactor LVR 15 (CV Řež, Czech Republic, reactor core configuration is shown in Figure 3) in vertical irradiation channels (each has 4 irradiation slots).

Terbium-161 was produced in positions H5/2, H5/3, H6/2 or H6/3. The neutron fluxes achievable in these channels are listed in Table 8 and the calculated activity of ^161^Tb from 30 mg of enriched Gd_2_O_3_ target (98.2 ± 0.1% ^160^Gd) for each position is shown in Figure 4.

Time of irradiation varied from circa 59 to 421 h. The targets are marked chronologically and with the serial number of ampoules in the given irradiation. Parameters of irradiation and prepared targets are listed in Table 9.

Irradiated targets were transported to the Faculty of Nuclear Sciences and Physical Engineering (FNSPE) for the separation and purification of ^161^Tb.

### 4.2. Separation and Purification Process

The general procedure was as follows: the quartz ampoule was crushed, the irradiated target material was dissolved in concentrated HNO_3_ (with heating), evaporated to dryness, and dissolved in ultrapure water.

A glass chromatography column (Econo-Column^®^, Bio-Rad, Prague, Czech Republic) of 150 × 5 mm dimension (3 mL) was filled with the cation exchange resin Dowex 50 W × 8 (H^+^) (200–400 mesh) in NH_4_^+^ form. The column was loaded with dissolved target and washed with 2 mL of ultrapure water. After loading, the column was connected to a low pressure peristaltic pump (PCD 1084, Peristaltická čerpadla a dávkovače Ing. Jindřich Kouřil, Kyjov, Czech Republic) and washed with 0.13 M α-HIBA solution (pH 4–4.5) to elute impurities and ^161^Tb, then with 0.5 M α-HIBA solution (pH 4–4.5) to recover the remaining gadolinium. The flow rate was mostly 0.2–0.25 mL/min. The volume of α-HIBA in both concentrations varied depending on target mass, usually between 80 and 170 mL for 0.13 M α-HIBA and between 10 and 20 mL for 0.5 M α-HIBA. Recovered gadolinium was recycled and prepared for another irradiation. Fractions were collected with SFC 90 fraction collector (ÚOCHB AV ČR, v. v. i., Prague, Czech Republic).

Terbium-161 containing fractions obtained after the separation process were mixed with an appropriate amount of concentrated HCl to adjust the pH to 1. The acidified fractions were passed through the small column (1 mL Rezorian^TM^ tube) filled with 0.5 mL of Dowex 50 W × 8 (H^+^) (200–400 mesh) in H^+^ form. The column was washed with 9 mL of 1 M HCl solution to eliminate α-HIBA and then with 4 M HCl solution until all ^161^Tb was washed out in the form of [^161^Tb]TbCl_3_ in several fractions (usually 5 to 10 mL). These fractions were joined, evaporated and reconstituted in 0.05 M HCl solution.

### 4.3. Radiometric Measurements

The radioactivity of all samples was measured by CRC-55tW detector. The radionuclide purity of prepared ^161^Tb was verified by gamma spectrometry on an HPGe detector (ORTEC^®^ DSPEC jr. 2.0 ™). The calibration was performed using a set of standards (^241^Am, ^152^Eu, ^133^Ba) provided by the Czech Institute of Metrology. At the beginning of the experiments, all fractions from separation were measured. After the method establishment, only ^161^Tb containing fractions were verified before purification. Terbium-160 was measured after the sufficient decay of ^161^Tb.

### 4.4. Specific Activity Determination—ICP-MS Analyses, DOTA Labelling

The presence of stable impurities in samples taken after the purification process was measured by the ICP-MS method. Analysis of ICP-MS was performed on an Agilent system 7500 in multi-element mode without a collision program. Samples were diluted with 5% nitric acid (15–30 µL of purified ^161^Tb solution into 3 mL) and were subsequently injected through an autosampler into nebulizer during 60 s. Multi-Element Quality Control Standards 1 and 2 (10 mg.L^−1^ in 2–5% nitric acid, series F96350 and 163665 Aristar) were used as calibration standards. Data were processed in Mass Hunter 1.01. Stable terbium was determined by its isotope ^159^Tb, which is its only stable isotope. All stable isotopes of gadolinium were measured and various isotopes of dysprosium were measured (^161^Dy, ^162^Dy, ^163^Dy, ^164^Dy). Other elements were determined by calculation from measurements of one of their stable isotopes. The determined concentrations of stable impurities are listed in Table 11.

For the evaluation of the quality of the stock solution of purified ^161^Tb in 0.05 M HCl, a procedure of radiolabelling of DOTA chelator with ^161^Tb followed by thin layer chromatography (TLC), as described by Gracheva et al. (2019) [22], was adopted with some modifications. Terbium-161 in 0.05 M HCl (2 MBq) was mixed with DOTA solution in different molar ratios in 0.5 M sodium acetate (pH 4.5). The total volume of reaction mixture was 25 µL. This mixture was heated to 95 °C for 20 min. This experiment was repeated 6 times during 14 days after the end of separation (EOS). The aliquots of each solution were deposited on TLC silica gel 60F_254_ plates (stationary phase) and eluted in mixture of 10% ammonium acetate and methanol (mobile phase, ration 1:1). Radiochromatograms were subsequently acquired on AR2000 (Bioscan) TLC reader with a mixture of argon-methane-isobutane as counting gas. The results from target 8_2 are presented in Figure 7.

## 5. Conclusions

A method of high activity (up to 22 GBq) n.c.a. ^161^Tb production in lab scale environment and subsequent separation and purification from enriched ^160^Gd_2_O_3_ target material were developed with the use of cation exchange resin Dowex 50 W × 8(H^+^), for both separation and purification parts of the process. The final ^161^Tb product was obtained in ^161^TbCl_3_ form in quantity and quality suitable for radiolabelling with potential use in preclinical research. In the future, we plan to continue in the optimisation of the process, mainly the purification step. We also plan to automate the method, to further reduce unnecessary radiation exposure.

We were able to repeatedly successfully produce pure ^161^Tb in activities up to 22 GBq (EOI), with specific activity up to 4.1 GBq/μg (of Tb) (EOI). The purity of the final product was verified by several methods (γ-spectroscopy, ICP-MS, DOTA radiolabelling). The prepared ^161^Tb meets high standards of radiochemical and radionuclidic purity, enabling its use for labelling both small molecules (e.g., PSMA), monoclonal antibodies and proteins (e.g., eFGF1-^161^Tb [30]), in cell-culture based experiments as well as in vivo models.

Based on these results and our experience, we have identified common issues in ^161^Tb production and focused on their practical aspects. Even at the laboratory scale, it is possible to produce up to 20 GBq of ^161^Tb which is fully sufficient for research purposes. While commercial companies employ more sophisticated processes, and over time it may become easier to purchase ^161^Tb from multiple sources rather than to produce it in-house, the available quantities may still be insufficient to meet the growing demand for (pre)clinical applications and research.

The aim of this work, beyond ^161^Tb production, was to highlight the minor yet critical issues that can arise during production. Published data, do not always reflect real conditions, and reproducibility is sometimes poor. Therefore, we focused on small details that are crucial from a practical standpoint and may even surprise experienced radiochemists who are not familiar with lanthanides chemistry.

## Figures and Tables

**Table 1:** Isotopes of terbium with potential use in nuclear medicine—half-life (T_1/2_), type and energy of decay and potential application [2,3].

Nuclide	T_1/2_	Type of Decay	$E_{\alpha }$ [MeV]	$\bar{E_{\beta }}$ [MeV]	$E_{\gamma }$ [keV] (I_γ_ [%])	Application
^149^Tb	4.12 h	α (16.7%) EC + β^+^ (83.3%)	3.967 (I_α_ = 16.7%)	0.730 (I_β+_ = 7.1%)	165.0 (26.4) 352.2 (29.4) 388.6 (18.4) 652.1 (16.2)	Alpha therapy PET imaging
^152^Tb	17.48 h	EC + β^+^ (100%)	-	1.140 (I_β+_ = 20.3%)	271.1 (9.5) 344.3 (63.5) 586.3 (9.2) 778.9 (5.5)	PET imaging
^155^Tb	5.32 d	EC (100%)	-	-	86.55 (32.0) 105.3 (25.1) 180.1 (7.5) 262.3 (5.3)	SPECT imaging
^161^Tb	6.95 d	β^−^ (100%)	-	0.154 (I_β_^−^ = 101%)	25.6 (23.2) 48.9 (17.0) 74.6 (10.2)	Beta/Auger therapy SPECT imaging

**Table 2:** Clinical trials involving ^161^Tb (in the time of writing) [13,14,15,16,17,18,19]. Notes: RLT = radioligand therapy; Obs. [PR] = Observational [Patient Registry]; Int. = Interventional; est. = estimated.

Clinical Trial ID	Title	Conditions	Intervention/Treatment; Drug:	Study Type	Phase	Enrollment
NCT04833517	REALITY Study: Analysis of a Prospective REgistry to Assess Outcome and Toxicity of Targeted RadionucLide TherapY in Patients With mCRPC in Clinical Routine	Prostate Cancer Metastatic; Castration-resistant Prostatic Cancer; Advanced Prostate Carcinoma	[^177^Lu]Lu-PSMA RLT; [^225^Ac]Ac-PSMA RLT; Tandem [^177^Lu]Lu-PSMA/[^225^Ac]Ac-PSMA RLT; [^223^Ra]RaCl_2_; [^153^Sm]Sm-EDTMP; [^90^Y]Y-microspheres; [^161^Tb]Tb-PSMA RLT	Obs. [PR]		500 (est.)
NCT05521412	VIOLET Study: EValuation of radIOLigand Treatment in mEn With Metastatic Castration-resistant Prostate Cancer With [^161^Tb]Tb-PSMA-I&T	Prostate Cancer; Metastatic Castration-resistant Prostate Cancer	[^161^Tb]Tb-PSMA-I&T	Int.	Phase 1 Phase 2	30 (actual)
NCT05359146	Beta Plus Study: Combined Beta-Plus Auger Electron Therapy Using a Novel Somatostatin Receptor Subtype 2 Antagonist Labelled With ^161^Terbium (^161^Tb-DOTA-LM3)	Neuroendocrine Neoplasias (NENs); Gastroenteropancreatic Neuroendocrine Tumour (GEP-NET)	[^161^Tb]Tb-DOTA-LM3; [^177^Lu]Lu-DOTATOC	Int.	Early Phase 1	16 (est.)
NCT06343038	Targeted Radionuclide Therapy in Metastatic Prostate Cancer Using a New PSMA Ligand Radiolabelled With Terbium-161 (161Tb-SibuDAB)—Dose Identification/Escalation Phase Ia/b Study	Castration-resistant Prostate Cancer	[^161^Tb]Tb-SibuDAB; [^177^Lu]Lu-PSMA-I&T	Int.	Phase 1	25 (est.)
NCT06827080	Study on the Safety, Tolerability, and Preliminary Efficacy of ^161^Tb-NYM032 in Patients with Metastatic Castration-Resistant Prostate Cancer	Metastatic Castration-resistant Prostate Cancer, MCRPC Cancer	[^161^Tb]Tb-NYM032	Int.	Phase 1 Phase 2	15 (est.)
NCT07208240	TbeforePROST Trial: Tb-PSMA-I&T Radionuclide Before Radical Prostatectomy in Patients With Locally Advanced Prostate Cancer	High Risk Prostate Cancer	Tb-PSMA-I&T (Tb-PSMA)	Int.	Phase 1 Phase 2	20 (est.)
NCT07259213	RAD402: A Study of Terbium-161 (161Tb)-RAD402 in Participants With CRPC	Castration-resistant Prostate Cancer	[^161^Tb]Tb-RAD402	Int.	Phase 1 Phase 2	73 (est.)

**Table 3:** Comparison of several cation exchange resins used for separation of ^161^Tb from ^160^Gd enriched targets.

Resin	Form	Particle Size	Eluent	Ref.
BioRad Aminex A6	NH_4_^+^	17.5 μm	^161^Tb: 0.13 M α-HIBA (pH 4.5) Gd: 0.5 M α-HIBA	[8]
Sykam macroporous cation exchange	NH_4_^+^	12–22 μm	^161^Tb: 0.13 M α-HIBA (pH 4.5) Gd: 0.175 M α-HIBA	[22]
BioRad AG 50 W × 8	H^+^	200–400 mesh (dry), 63–150 µm (wet bead size)	^161^Tb: 0.07 M α-HIBA (pH 4.75) Gd: 6.0 M HNO_3_	[24]

**Table 4:** Comparison of several extraction resins used for separation of ^161^Tb from ^160^Gd targets.

Separation Step	Eluent—c(HNO_3_)	Purification/Concentration Step—Resin and Eluent	Ref.
LN resin	Gd: 0.8 N, ^161^Tb: 3.0 N	none	[23]
LN2 resin	Gradient elution: 0.25 M, 0.45 M, 1.0 M	DGA resin, 0.05 M HCl	[25]
TK212 resin (1st column) TK211 resin (2nd column)	Gd: 0.2 M, ^161^Tb and ^161^Dy: 0.5 M rest of Gd: 0.5 M, ^161^Tb: 0.75 M	TK221, 0.05 M HCl	[26]

**Table 5:** Comparison of fresh and recycled gadolinium target material. Theoretical specific activity for ^161^Tb is 4.3 GBq/μg (of Tb).

	Fresh (as Purchased)	Recycled
Preparation of the target before irradiation	Oxide form—“ready to use”; Better prediction of the outcome	Needs to be processed before use—possible target material loss and contamination
Handling during target preparation	Easier—non-radioactive material	Appropriate handling required—radioactive material with long-live products
Stable impurities	More stable impurities *	Less stable impurities and higher specific activity of ^161^Tb (gradual ^158^Gd burning, previous separations); possible presence of carbides (from α-HIBA residues)
Radioactive impurities	None	Long-live radioactive isotopes (^153^Gd) and possibly radioactive impurities (^152^Eu, ^154^Eu)
Specific activity of the final ^161^Tb product [MBq/μg (of Tb)]	300–1100	4000–4100
Availability	Currently limited amount available	Self-sufficient source when repeatedly recycled (with regards to gradual loss of the material)

* See the isotopic composition certificate from the producer (Tables 12 and 13).

**Table 6:** Comparison of gadolinium target in oxide and nitrate form.

	Gd_2_O_3_	Gd(NO_3_)_3_ · 6 H_2_O
Preparation time	Short—only sealing of the appropriate amount of material in quartz ampoule	Long—converting from oxide, sealing of the appropriate amount of material in quartz ampoule, higher risk of target contamination
Ampoule filling issues	Electrostatic repulsion of Gd_2_O_3_	Hygroscopic material
Scalability	High	Low
Risk of ampoule failure and target loss	Low	High
Processing of the irradiated target	More complicated—need to use concentrated acids, evaporation, redissolution, heating; risk of contamination of the surrounding area and the dissolved target	Significantly easier than oxide target—dissolution in less concentrated acid without heating

**Table 7:** Comparison—two methods of opening of the irradiated ampoule.

	Controlled Cutting	Crushing
Use when the target material	remains on the bottom of the ampoule	is already spread throughout the ampoule, or the inner diameter is too narrow
Target handling	Manual operation	Automated and remote controlled operation
Volume of the dissolved target *	Smaller (0.5–1 mL)	Larger (2–5 mL)

* The volume depends also on the form of the target (oxide or nitrate), because of the solubility.

**Table 8:** Calculated neutron fluxes in the vertical irradiation channels at positions used for ^161^Tb production in the LVR-15 research reactor. The “Total” value represents the sum of the first three entries in each column, excluding the fourth.

Energy	Neutron Flux (n.cm^−2^.s^−1^)			
	H5/2	H5/3	H6/2	H6/3
<0.5 eV	6.97 × 10^+13^	7.03 × 10^+13^	6.68 × 10^+13^	5.83 × 10^+13^
0.5 eV–0.1 MeV	4.24 × 10^+13^	3.83 × 10^+13^	3.71 × 10^+13^	3.59 × 10^+13^
0.1–20 MeV	3.36 × 10^+13^	3.02 × 10^+13^	3.47 × 10^+13^	3.36 × 10^+13^
>1.0 MeV	1.59 × 10^+13^	1.44 × 10^+13^	1.72 × 10^+13^	1.67 × 10^+13^
**Total**	1.46 × 10^+14^	1.39 × 10^+14^	1.39 × 10^+14^	1.28 × 10^+14^

**Table 9:** Production runs of ^161^Tb with targets in the form of oxide or nitrate, prepared from fresh or. Note: A_EOI_ DT = Activity of dissolved target (related to the end of irradiation).

Target	Target Mass [mg]	Mass ^160^Gd [mg]	Form	Target Condition	Irradiation Time [h]	Position	Reactor Power [MW]	A_EOI_ DT [GBq]
1_1	46.4	39.6	oxide	fresh	145.50	H5/3	9.40	5.6
1_2	106.4	36.8	nitrate	fresh				6.4
2_1	141.1	48.8	nitrate	fresh	162.90	H5/3	9.26	7.9
2_2	119.7	41.4	nitrate	fresh				6.0
3_1	43.4	15.0	nitrate	recycled	59.20	H5/3	9.70	1.3
3_2	55.9	47.7	oxide	recycled				3.2
4_1	144.0	49.8	nitrate	recycled	222.70	H6/3	9.69	11.0
4_2	76.0	26.3	nitrate	recycled				3.1
5_1	12.5	10.7	oxide	fresh	196.98	H5/2	9.68	11.1
5_2	12.5	10.7	oxide	fresh				
5_3	12.5	10.7	oxide	fresh				
5_4	12.5	10.7	oxide	fresh				
6_1	12.5	10.7	oxide	fresh	266.08	H6/2	9.67	11.7
6_2	12.5	10.7	oxide	fresh				
6_3	12.5	10.7	oxide	fresh				
6_4	12.5	10.7	oxide	fresh				
7_1	48.5	41.4	oxide	recycled	287.90	H5/2	9.70	11.8
8_1	98.3	84.0	oxide	fresh	286.40	H5/3	9.67	21.8
8_2	49.8	42.5	oxide	recycled				12.0
9_1	59.5	50.8	oxide	fresh	239.00	H5/2	9.69	15.3
10_1	65.0	55.5	oxide	recycled	240.00	H5/2	9.69	10.7
11_1	98.3	84.0	oxide	recycled	314.00	H5/2	9.68	23.7
12_1	49.0	41.9	oxide	fresh	421.52	H5/2	9.68	15.8

**Table 10:** Parameters of separations performed on a glass chromatography column (150 × 5 mm dimension) filled with Dowex 50 W × 8 (H+) (200–400 mesh) in NH_4_^+^ form with flow rate 0.25 mL/min for target 12_1 and 0.20 mL/min for all other targets, activity of obtained pure ^161^Tb (A_EOI_), yield of pure ^161^Tb (Y; [^161^Tb]TbCl_3_ in 0.05 M HCl) and specific activity (A_m_) of ^161^Tb solutions. Notes: Targets 6_1, 6_2, 6_3 and 6_4 were combined after irradiation and processed together. Target 11_1 (98.3 mg), only a half of the irradiated target was processed.

Target	A_EOI_ [GBq]	Y [%]	A_m_ [MBq/μg]	^160^Gd Content [μg/GBq]
6_(1–4)	11.2	99.5	1200	192.67
7_1	9.9	93.7	4200	0.0076
8_1	22.1	90.5	4100	0.8568
8_2	11.4	89.8	4000	0.0727
9_1	14.7	86.8	500	0.0081
10_1	10.4	85.7	4100	0.0012
11_1	13.6	88.7	4000	0.0491
12_1	15.8	88.6	400	0.0127

**Table 11:** Concentration of stable impurities (*c*) in samples from purified ^161^Tb solution. The value “<LOD” means that the measured concentration of the respective isotopes was below the detection limit of the ICP-MS.

Target		^159^Tb	Gd	Cr	Ni	Cu	Zn	Nd	Sm	Eu	Er
c (μg/mL)	6_(1–4)	1.219	974.975	0.170	<LOD	<LOD	0.786	0.099	0.029	0.032	0.025
	7_1	0.018	0.026	0.609	<LOD	<LOD	<LOD	0.031	0.007	0.002	0.010
	8_1	0.603	160.409	0.229	<LOD	<LOD	<LOD	0.037	0.015	0.016	<LOD
	8_2	0.118	0.663	0.138	<LOD	<LOD	<LOD	0.062	0.044	0.012	0.032
	9_1	10.285	16.115	130.684	43.450	5788.145	14.028	1.613	7.405	2.127	5.806
	10_1	0.135	0.081	0.091	<LOD	37.025	<LOD	<LOD	<LOD	0.038	0.034
	11_1	0.161	0.551	<LOD	<LOD	40.623	<LOD	<LOD	<LOD	0.019	0.011
	12_1	88.268	12.668	104.547	28.825	4310.207	17.642	1.291	1.481	1.276	3.313

**Table 12:** Target material—Isotopic composition for 500 mg of Gd_2_O_3_ with 98.2 ± 0.1% ^160^Gd enrichment (ISOFLEX USA).

Isotope	^152^Gd	^154^Gd	^155^Gd	^156^Gd	^157^Gd	^158^Gd	^160^Gd
Content (%)	<0.001	0.01	0.18	0.36	0.25	1	98.2 ± 0.1

**Table 13:** Target material—Chemical admixtures for 500 mg of Gd_2_O_3_ with 98.2 ± 0.1% ^160^Gd enrichment (ISOFLEX USA).

Element	Content (%)	Element	Content (%)	Element	Content (%)
K	<0.005	Si	<0.005	Nd	<0.0001
Na	<0.002	Cr	<0.0005	Sm	0.0013
Ca	<0.005	Ni	<0.0001	Eu	<0.0001
Mg	<0.0003	Cu	<0.0001	Tb	<0.0002
Fe	<0.005	Pb	0.0013	Dy	<0.0001
Al	<0.0003	Sb	<0.0001	Er	<0.0001

## Data Availability

The data presented in this study are available upon request from the corresponding author. The data are not publicly available because they contain proprietary technical details related to commercially sensitive production processes and quality control procedures.

## Abbreviations

The following abbreviations are used in this manuscript:

[^161^Tb]Tb-RAD402	anti-KLK3 monoclonal antibody radiolabelled with the radionuclide ^161^Tb
<LOD	below the limit of detection
CT	computing tomography
DOTA	2,2′,2′′,2′′′-(1,4,7,10-Tetraazacyclododecane-1,4,7,10-tetrayl)tetraacetic acid
DOTA-LM3	DOTA-p-Cl-Phe-cyclo(d-Cys-Tyr-d-4-amino-Phe(carbamoyl)-Lys-Thr-Cys)d-Tyr-NH_2_
DOTATOC	(DOTA^0^-Phe^1^-Tyr^3^) octreotide; Edotreotide
DTPA	diethylenetriaminepentaacetic acid
EDTMP	Ethylenediamine tetra(methylene phosphonic acid)
eFGF1-^161^Tb	Engineered FGF1-DOTA-Tb [^161^Tb] for targeting FGFRs
FGFRs	fibroblast growth factor receptors
EOI	end of irradiation
EOS	end of separation
ICP-MS	inductively coupled plasma mass spectrometry
mAb	molecular antibody
PET	Positron emission tomography
PSMA	Prostate-specific membrane antigen
PSMA-I&T	Prostate-specific membrane antigen for imaging & therapy
RLT	radioligand therapy
SibuDAB	(S)-ibuprofen-diaminobutyric acid-PSMA
SPECT	Single-photon emission computing tomography
TAT	targeted alpha therapy
